# Physicians’ experiences with euthanasia: a cross-sectional survey amongst a random sample of Dutch physicians to explore their concerns, feelings and pressure

**DOI:** 10.1186/s12875-019-1067-8

**Published:** 2019-12-17

**Authors:** Kirsten Evenblij, H. Roeline W. Pasman, Johannes J. M. van Delden, Agnes van der Heide, Suzanne van de Vathorst, Dick L. Willems, Bregje D. Onwuteaka-Philipsen

**Affiliations:** 1 Department of Public and Occupational Health, VUmc Expertise Center for Palliative Care, Amsterdam Public Health Research Institute, Amsterdam University Medical Centers, Vrije Universiteit, Amsterdam, The Netherlands; 2 UMC Utrecht, Julius Center for Health Sciences and Primary Care, Department of Medical Humanities, Utrecht, The Netherlands; 3 Department of Public Health Erasmus MC, University Medical Center Rotterdam, Rotterdam, The Netherlands; 4 Department of General Practice, section Medical Ethics, Amsterdam Public Health Research Institute, Amsterdam University Medical Centers, University of Amsterdam, Amsterdam, The Netherlands

## Abstract

**Background:**

Physicians who receive a request for euthanasia or assisted suicide may experience a conflict of duties: the duty to preserve life on the one hand and the duty to relieve suffering on the other hand. Little is known about experiences of physicians with receiving and granting a request for euthanasia or assisted suicide. This study, therefore, aimed to explore the concerns, feelings and pressure experienced by physicians who receive requests for euthanasia or assisted suicide.

**Methods:**

In 2016, a cross-sectional study was conducted. Questionnaires were sent to a random sample of 3000 Dutch physicians. Physicians who had been working in adult patient care in the Netherlands for the last year were included in the sample (*n* = 2657). Half of the physicians were asked about the most recent case in which they refused a request for euthanasia or assisted suicide, and half about the most recent case in which they granted a request for euthanasia or assisted suicide.

**Results:**

Of the 2657 eligible physicians, 1374 (52%) responded. The most reported reason not to participate was lack of time. Of the respondents, 248 answered questions about a refused euthanasia or assisted suicide request and 245 about a granted EAS request. Concerns about specific aspects of the euthanasia and assisted suicide process, such as the emotional burden of preparing and performing euthanasia or assisted suicide were commonly reported by physicians who refused and who granted a request. Pressure to grant a request was mostly experienced by physicians who refused a request, especially if the patient was ≥80 years, had a life-expectancy of ≥6 months and did not have cancer. The large majority of physicians reported contradictory emotions after having performed euthanasia or assisted suicide.

**Conclusions:**

Society should be aware of the impact of euthanasia and assisted suicide requests on physicians. The tension physicians experience may decrease their willingness to perform euthanasia and assisted suicide. On the other hand, physicians should not be forced to cross their own moral boundaries or be tempted to perform euthanasia and assisted suicide in cases that may not meet the due care criteria.

## Background

Physicians are trained to preserve and prolong the life of patients by curing illnesses. However, in case of advanced illness, cure may no longer be possible and the relief of suffering becomes the primary aim of care. Palliative care is an approach aimed at improving the quality of life of patients facing life-limiting illness through the prevention and relief of suffering [1]. In some cases, however, the patient may develop the desire to hasten death [2–4].

In the Netherlands, more than 12,000 patients requested their physician for euthanasia or assisted suicide (EAS) in 2015 [5]. The Netherlands is one of the few countries in the world with a legal system that regulates the practice of EAS in case of unbearable suffering [6]. As the Dutch euthanasia act is mainly based on the principle of compassion of the physician and EAS is still considered to be an exceptional medical act, physicians cannot be forced to perform EAS. However, physicians may choose to perform EAS as a deed of beneficence if the due care criteria are met and if the physician is willing to grant the request [7, 8]. Dutch physicians are generally open to performing EAS. Approximately 55% of all explicit, concurrent EAS requests were granted in 2015. In 2016, 57% of the physicians had ever performed EAS in 2016 and another 24% who had never performed EAS found it conceivable to do so in the future [9].

Physicians who receive a request for EAS may experience a conflict of duties: the duty to preserve life on the one hand and the duty to relieve suffering on the other hand. Although, the frequency of EAS requests and the involvement of physicians in the practice of EAS has been studied repeatedly, little is known about experiences of physicians with receiving and granting an EAS request, such as the emotional impact or perceived pressure. In 1995, a study demonstrated that 72% of the Dutch physicians experienced feelings of discomfort after performing EAS: the act was mostly referred to as a burdensome or a heavy responsibility [10]. Another, more recent study (2011), showed that in the previous 5 years 49% of the physicians had at least once experienced pressure from the patient to grant the request [11]. The source of the distress possibly lies with (aspects of) the EAS procedure, such as assessing whether the patient meets the due care criteria [12], performing EAS, or the administrative burden of reporting the act. Given that the large majority of Dutch physicians will be confronted with an EAS request at some point in their career [9], it is important to get insight in the experiences of physicians with EAS. The purpose of this study was to explore the experiences of physicians with receiving requests for and performing EAS. The following research questions are addressed in this paper: i) Do physicians have concerns about the euthanasia process and, if so, about what specific aspects? ii) Do physicians feel pressured to provide euthanasia when they receive a request for euthanasia and, if so, by whom? iii) What factors are associated with experiencing pressure? iv) What feelings are experienced by physicians who granted a request and do they seek support? and v) What factors are associated with physicians experiencing performing euthanasia as burdensome?

## Methods

### Design and participants

In the context of the third evaluation of the Dutch euthanasia act, a cross-sectional study was conducted to explore the experiences of physicians with receiving requests for and performing EAS. A questionnaire was sent to the home or work addresses of a random sample of 1100 general practitioners, 400 elderly care physicians, 1000 medical specialists (working in hospital) and 500 psychiatrists. Addresses were obtained from a national databank of registered physicians (IMS Health). Physicians who had been working in adult patient care in the Netherlands for the last year were included in the sample. This study did not require review by an ethics committee under the Dutch Medical Research Involving Human Subjects Act, since it did not involve imposing any interventions or actions [13].

### Data collection and questionnaire

All selected physicians received a 12-page questionnaire on paper. The questionnaire was similar to the one that was used in the second (2011) and first (2005) evaluation of the euthanasia act [11, 14]. Besides the questionnaire, selected physicians also received a response card with the respondent’s name and address and the option to indicate whether they wished to participate in the study, and if not, why not. The questionnaire and the response card were to be returned in separate envelopes. Thereby anonymity was guaranteed without precluding the possibility of sending of two reminders to non-respondents. Informed consent was assumed on return of the survey. Data were collected between May and September 2016.

The questionnaire contained questions about the most recent case in which the physicians had received a request for EAS from a patient who did not have cancer. It was explained that if the physician had only received requests from patients with cancer, he or she could describe this case. The rationale for this was to retrieve as many non-cancer patients as possible as the majority of euthanasia requests are from patients with cancer. In the questionnaire, physicians were asked for patient characteristics, their concerns regarding (aspects of) the EAS process, and whether they had experienced pressure from patients, relatives or others to either grant or refuse the request. Half of the physicians were asked to answer questions about the most recent case in which they refused a request, the other half were asked to answer questions about the most recent case in which they granted a request. The questionnaire on granted requests contained additional questions on the feelings experienced by physicians who performed EAS, support received to cope with performing EAS, and the occurrence of complications or unexpected events during the performance of EAS. As it was expected that few psychiatrists had granted an EAS request, psychiatrists were asked to fill out questions on the most recent case in which they refused a request and the most recent case in which they granted a request, if any.

The questionnaire also included a general section on respondent characteristics including as age, gender, specialty. Besides, physicians were asked how many requests they had received in the last year and whether they felt generally pressured by society to grant EAS requests.

### Statistical analyses

Statistics were carried out in IBM SPSS version 22 (IBM Analytics). Only respondents who had answered questions about a refused or granted request were included in the analysis. Frequencies were calculated to describe patient and physician characteristics and experiences with the EAS process: concerns with regard to the EAS process, pressure from the patient to grant the request, feelings experienced by physicians performing EAS and support sought to deal with it, and the occurrence of complications or adverse events. Differences between refused and granted cases were analysed using Chi-square tests or a Fisher’s Exact Test in case of small cell sizes (> 20% cells have expected count < 5).

To analyse which factors were associated with i) perceived pressure and ii) with a burdensome experience, univariable logistic regression analyses were performed. The analyses to identify factors associated with perceived pressure from the patient to grant the request were stratified for the outcome of the request: granted vs refused. Patient factors entered in the models were: gender (male/female), age (< 65, 65–79, ≥80), cancer (yes/no) the presence of a psychiatric disorder (yes/no), life-expectancy (< 1 month, 1–5 months, ≥6 months), possibility of communication (good/ less than good), and opinions of relatives regarding the request (supporting/not supporting). Physician factors entered in the models were: specialty (general practitioner, elderly care physician, medical specialist, psychiatrist), gender (male/female) age (< 40, 40–54, ≥55), number of requests received in the last year (0, 1, 2, ≥3). The univariable logistic regression analyses to identify factors associated with perceiving EAS as a burdensome experience were performed on a subset of the sample: physicians who had granted a request. Besides the patient and physician factors described above, the following factors were entered to the model: pressure experienced to perform EAS as soon as possible (yes/no), concerned about administering lethal drugs (yes/no), the occurrence of complications or adverse events (yes/no), and seeking support after the performance of EAS (yes/no). Odds ratios (ORs) with 95% Confidence Intervals (CIs) were calculated. A *p*-value < 0.05 was considered statistically significant.

## Results

Of the 3000 sampled physicians, 343 physicians did not meet the inclusion criteria. Of the 2657 eligible physicians, 1374 (52%) responded. Two hundred one out of 1283 non-responders returned a response card reporting the reason for non-response. The most reported reason not to participate was lack of time (*n* = 148). Of the 1374 respondents, 248 filled out questions about a refused request and 245 filled out questions about a granted request. The physicians who did not fill out questions about a refused or granted request had either never received a request for EAS, never granted or never refused a request, or did not provide information on a request for unknown reasons.

### Concerns of physicians who received an EAS request

Of the physicians who reported about a refused request, 48.0% dreaded the emotional burden of performing EAS compared to 58.3% of the physicians who reported about a granted request (Table 1, *p* = 0.026). Amongst physicians who reported about a refused EAS request, concerns about assessing whether the due care criteria were met, dealing with the relatives of the patient, and the reactions of other care providers were more frequently reported compared to physicians who reported about a granted request (38.5% vs 21.7%, *p* < 0.001; 28.3% vs 11.4%, p < 0.001; and 18.3% vs 5.4%, p < 0.001). Of the physicians who reported about a granted request, 41.4% dreaded the administrative burden of notifying the unnatural death compared to 24.4% of the physicians who reported about a refused request (*p* < 0.001).

**Table 1:** Concerns in the most recent case of a euthanasia request

	Request refused	Request granted	*p*-value
	Total *N* = 248^a^	Total *N* = 245^a^	
	*N* (%)	*N* (%)	
*I was dreading*			
The emotional burden of performing EAS	107 (48.0%)	140 (58.3%)	0.026
The emotional burden of preparing EAS	106 (45.9%)	130 (54.2%)	0.072
Assessing whether the due care criteria have been met	90 (38.5%)	52 (21.7%)	< 0.001
The time the decision-making process would take	74 (31.6%)	66 (27.8%)	0.370
Dealing with relatives of the patient	64 (28.3%)	27 (11.4%)	< 0.001
Waiting for the judgement of the euthanasia review committees	56 (25.5%)	63 (26.4%)	0.825
The administration of lethal drugs	47 (25.0%)	77 (32.5%)	0.092
The administrative burden of notifying the unnatural death	54 (24.4%)	99 (41.4%)	< 0.001
The reactions of other care providers	42 (18.3%)	13 (5.4%)	< 0.001

^a^Missings: Refused cases missing cases varied between 14 and 60 (5.6–24.2%), Granted cases missing cases varied between 5 and 8 (2.0–3.3%)

### Pressure experienced by physicians who received an EAS request

Of all physicians in the sample, 44.4% felt pressure by society in general to grant EAS requests, 40.8% did not and 14.8% answered neutral. Of the physicians who reported about a refused request, 60.3% felt pressured by the patient to grant the request compared to 13.2% of the physicians who reported about a granted request (Table 2, *p* < 0.001). Amongst those who refused the request were also more physicians who felt pressured by relatives of the patient to grant the request (31.7% vs 6.2% p < 0.001) and by the patient and or his/her relatives to perform EAS as soon as possible (35.5% vs 22.6%, *p* = 0.002). Few physicians reported to have experienced pressure from colleagues or the management of their institution to either grant or refuse the request.

**Table 2:** Pressure in the most recent case of a euthanasia request^a^

	Request refused	Request granted	*p*-value
	Total *N* = 248	Total *N* = 245^a^	
	*N* (%)	*N* (%)	
*I felt pressured by…*			
the patient to grant the request	146 (60.3%)	32 (13.2%)	< 0.001
the patient or his/her relatives to perform EAS as soon as possible	86 (35.5%)	55 (22.6%)	0.002
relatives of the patients to grant the request	77 (31.7%)	15 (6.2%)	< 0.001
colleagues to refuse the request	9 (3.7%)	5 (2.1%)	0.275
relatives of the patient to refuse the request	8 (3.3%)	5 (2.1%)	0.399
colleagues to grant the request	7 (2.9%)	1 (0.4%)	0.037^b^
the management to refuse the request	6 (2.5%)	1 (0.4%)	0.122^b^

^a^Missings: Refused cases missing cases varied between 5 and 6 (2.0–2.4%), Granted cases missing cases varied between 2 and 3 (0.8–1.2%)

^b^ 2 cells (50%) had expected count less than 5 hence a Fisher’s Exact test was performed

Table 3 shows that physicians who reported about a refused request of a patient aged 80 years or older were 2.03 times more likely to feel pressured by the patient to grant the request compared to physicians who reported about a refused request of a patient aged 64 years or younger (95% CI 1.08–3.78). Physicians who reported about a refused request of a patient with a life-expectancy of 6 months or more were 3.89 times more likely to feel pressured compared to physicians who reported about a refused request of a patient with a life-expectancy of less than 1 month (95% CI 1.51–9.99). If the patient had cancer, physicians were less likely to feel pressured compared to if the patient did not have cancer (OR 0.38 [0.19–0.74]). Medical specialists were less likely to feel pressured by the patient to grant the request compared to general practitioners (OR 0.35 [0.13–0.95]). Physicians aged 55 years or older were 2.90 times more likely [95% CI 1.27–6.62] to feel pressured compared to physicians who were aged 39 years or younger. Amongst physicians who granted the request, no significant associations were identified of patient and physician factors with perceived pressure to grant the request.

**Table 3:** Factors associated with perceived pressure (pressured by patient to grant the request)

	Refused cases		Univariable logistic regression	Granted cases		Univariable logistic regression
	No pressure	Pressure		No pressure	Pressure	
	*N* = 96	*N* = 146	OR (95% CI)	*N* = 211	*N* = 32	OR (95% CI)
Patient characteristics						
Gender						
Male	43 (46.2%)	55 (37.9%)	Reference	116 (55.0%)	12 (37.5%)	Reference
Female	50 (53.8%)	90 (62.1%)	1.41 (0.83–2.39)	95 (45.0%)	20 (62.5%)	2.04 (0.95–4.38)
Age (year)						
≤ 64	45 (46.9%)	48 (32.9%)	Reference	53 (25.2%)	11 (34.4%)	Reference
65–79	26 (27.1%)	44 (30.1%)	1.59 (0.84–2.99)	97 (46.2%)	11 (34.4%)	0.55 (0.22–1.34)
≥ 80	25 (26.0%)	54 (37.0%)	**2.03 (1.08–3.78)**	60 (28.6%)	10 (31.3%)	0.80 (0.32–2.04)
Cancer						
No	69 (72.6%)	126 (87.5%)		91 (43.3%)	19 (59.4%)	Reference
Yes	26 (27.4%)	18 (12.5%)	**0.38 (0.19–0.74)**	119 (56.7%)	13 (40.6%)	0.52 (0.25–1.12)
Presence psychiatric disorder						
No	61 (63.5%)	79 (54.1%)	Reference	197 (93.4%)	30 (93.8%)	Reference
Yes	35 (36.5%)	67 (45.9%)	1.48 (0.87–2.51)	14 (6.6%)	2 (6.3%)	0.94 (0.20–4.34)
Life-expectancy						
< 1 month	15 (15.8%)	7 (4.8%)	Reference	46 (21.8%)	4 (12.5%)	Reference
1–5 months	10 (10.5%)	12 (8.2%)	2.57 (0.75–8.78)	91 (43.1%)	10 (31.3%)	1.26 (0.38–4.25)
≥ 6 months	70 (73.7%)	127 (87.0%)	**3.89 (1.51–9.99)**	74 (35.1%)	18 (56.3%)	2.80 (0.89–8.78)
Communication possible						
Less than good (reasonable-barely)	42 (43.8%)	64 (44.1%)	Reference	16 (7.6%)	3 (9.4%)	Reference
Good	54 (56.3%)	81 (55.9%)	0.98 (0.59–1.66)	195 (92.4%)	29 (90.6%)	0.79 (0.22–2.89)
Opinions of relatives regarding the request						
Not supporting (neutral, divided, opposing)	54 (56.8%)	94 (64.8%)	Reference	10 (4.7%)	4 (12.5%)	Reference
Supporting	41 (43.2%)	51 (35.2%)	0.72 (0.42–1.21)	201 (95.3%)	28 (87.5%)	0.35 (0.10–1.19)
Physician characteristics						
Specialty						
General practitioner	42 (43.8%)	71 (49.3%)	Reference	151 (73.7%)	21 (67.7%)	Reference
Elderly care physician	18 (18.8%)	25 (17.4%)	0.82 (0.40–1.68)	28 (13.7%)	5 (16.1%)	1.28 (0.45–3.69)
Medical specialist	12 (12.5%)	7 (4.9%)	**0.35 (0.13–0.95)**	18 (8.8%)	3 (9.7%)	1.20 (0.33–4.42)
Psychiatrist	24 (25.0%)	41 (28.5%)	1.01 (0.54–1.90)	8 (3.9%)	2 (6.5%)	1.80 (0.36–9.04)
Gender						
Male	40 (42.6%)	64 (44.1%)	Reference	104 (49.8%)	19 (59.4%)	Reference
Female	54 (57.4%)	81 (55.9%)	0.94 (0.56–1.58)	105 (50.2%)	13 (40.6%)	0.68 (0.32–1.44)
Age (year)						
≤ 39	20 (20.8%)	16 (11.0%)	Reference	49 (23.2%)	5 (15.6%)	Reference
40–54	54 (56.3%)	79 (54.1%)	1.83 (0.87–3.84)	83 (39.3%)	19 (59.4%)	2.24 (0.79–6.39)
≥55	22 (22.9%)	51 (34.9%)	**2.90 (1.27–6.62)**	79 (37.4%)	8 (25.0%)	0.99 (0.31–3.21)
Number of explicit request received in the last year						
0	40 (41.6%)	54 (37.0%)	0.54 (0.26–1.14)	52 (24.6%)	10 (31.3%)	1.41 (0.48–4.19)
1	23 (24.0%)	34 (23.3%)	0.59 (0.26–1.34)	68 (32.2%)	9 (28.1%)	0.97 (0.32–2.92)
2	19 (19.8%)	23 (15.7%)	0.48 (0.20–1.15)	47 (22.3%)	7 (21.9%)	1.09 (0.34–3.50)
≥ 3	14 (14.6%)	35 (24.0%)	Reference	44 (20.9%)	6 (18.8%)	Reference

Missings: Refused cases missings varied between 6 and 10 (2.4–4.0%), Granted cases missings varied between 2 and 9 (0.8–3.7%). Bold indicates statistical significance (*p* < 0.05)

### Feelings reported by physicians who granted a request

Physicians were asked how they felt after having performed EAS. They could choose from a range of feelings that could be labelled as comfortable and uncomfortable feelings. Physicians could experience both at the same time. Of the 245 physicians who granted the EAS request, 66.7% reported comfortable feelings after having performed EAS (Table 4). This mostly concerned a feeling of satisfaction. Simultaneously, 80.0% reported feelings of discomfort: 49.6% of the physicians experienced it as burdensome, 45.8% as a heavy responsibility and 44.2% as emotional. The majority of the physicians (62.2%) did not seek support afterwards. Of the physicians who did (37.8%), most sought support from colleagues or from relatives.

**Table 4:** Physicians’ feelings after the most recent case in which they granted an EAS request and support sought to deal with this

	Total *N* = 245*
	*N* (%)
Feelings of “comfort”	
Satisfactory	145 (60.4%)
Relief	33 (13.8%)
Total	160 (66.7%)
Feelings of “discomfort”	
Burdensome	119 (49.6%)
Heavy responsibility	110 (45.8%)
Emotional	106 (44.2%)
Total	192 (80.0%)
Other feelings	
Unnatural	23 (9.6%)
Sought support	
No	150 (62.2%)
Yes, from	91 (37.8%)
colleagues/team	… 77 (31.4%)
from relatives (privekring)	… 60 (24.5%)
intervision	… 11 (4.5%)
professional support	… 1 (0.4%)
other	… 5 (2.0%)

*Missings varied between 4 and 5 (1.6–2%)

Physicians aged between 40 and 54 years were 2.18 times more likely [95% CI 1.11–4.28] to perceive performing EAS as burdensome compared to physicians aged 39 years or younger (Table 5). Physicians who had received no or only one explicit request in the last year were around 2.5 times more likely to perceive performing EAS as burdensome compared to physicians who had received 3 or more requests in the last year (OR 2.49 [1.15–5.39] and OR 2.58 [1.24–5.39]). Also physicians who felt pressured by the patient and/or his relatives to perform EAS as soon as possible (OR 1.91 [1.03–3.53]) and physicians who were concerned about administering the lethal drugs were more likely to experience burdensome feelings (OR 2.22 [1.27–3.87]).

**Table 5:** Factors associated with burdensome feelings after the most recent case of euthanasia^a^

	Not burdensome	Burdensome	Univariable logistic regression analysis
	*N* = 121	*N* = 119	
	*N* (%)	*N* (%)	OR (95% CI)
Patient characteristics			
Gender			
Male	60 (49.6%)	67 (56.3%)	Reference
Female	61 (50.4%)	52 (43.7%)	0.76 (0.46–1.27)
Age (year)			
≤ 64	31 (25.6%)	32 (26.9%)	Reference
65–79	52 (43.0%)	55 (46.2%)	1.03 (0.55–1.91)
≥ 80	38 (31.4%)	32 (26.9%)	0.82 (0.41–1.61)
Cancer			
No	52 (43.0%)	55 (46.6%)	Reference
Yes	69 (57.0%)	63 (53.4%)	0.86 (0.52–1.44)
Presence psychiatric disorder			
No	114 (94.2%)	111 (93.3%)	Reference
Yes	7 (5.8%)	8 (6.7%)	1.17 (0.41–3.35)
Life-expectancy			
< 1 month	26 (21.5%)	24 (20.2%)	Reference
1–5 months	55 (45.5%)	45 (37.8%)	0.89 (0.45–1.75)
≥ 6 months	40 (33.1%)	50 (42.0%)	1.35 (0.68–2.71)
Communication possible			
Less than good (reasonable-barely)	9 (7.4%)	10 (8.4%)	Reference
Good	112 (92.6%)	109 (91.6%)	0.88 (0.34–2.24)
Opinions of relatives regarding the request			
Not supporting (neutral, divided, opposing)	6 (5.0%)	7 (5.9%)	Reference
Supporting	115 (95.0%)	112 (94.1%)	0.84 (0.27–2.56)
Physician characteristics			
Specialty			
General practitioner	86 (72.9%)	86 (74.8%)	Reference
Elderly care physician	15 (12.7%)	17 (14.8%)	1.13 (0.53–2.41)
Medical specialist	14 (11.9%)	6 (5.2%)	0.43 (0.16–1.17)
Psychiatrist	3 (2.5%)	6 (5.2%)	2.00 (0.49–8.26)
Gender			
Male	61 (50.8%)	60 (50.8%)	Reference
Female	59 (49.2%)	58 (49.2%)	1.00 (0.60–1.66)
Age (year)			
≤ 39	32 (26.4%)	22 (18.5%)	Reference
40–54	40 (33.1%)	60 (50.4%)	**2.18 (1.11–4.28)**
≥ 55	49 (40.5%)	37 (31.1%)	1.10 (0.55–2.19)
Number of explicit request received in the last year			
0	25 (20.7%)	35 (29.4%)	**2.49 (1.15–5.39)**
1	31 (25.6%)	45 (37.8%)	**2.58 (1.24–5.39)**
2	33 (27.3%)	21 (17.6%)	1.13 (0.51–2.51)
≥ 3	32 (26.5%)	18 (15.1%)	Reference
I felt pressured by the patient and/or his relatives to perform EAS as soon as possible			
No	100 (82.6%)	85 (71.4%)	Reference
Yes	21 (17.4%)	34 (28.6%)	**1.91 (1.03–3.53)**
I was concerned about administering the lethal drugs			
No	91 (75.8%)	68 (58.6%)	Reference
Yes	29 (24.2%)	48 (41.4%)	**2.22 (1.27–3.87)**
Occurrence of complications or adverse events during the performance of EAS			
No	114 (95.8%)	110 (92.4%)	Reference
Yes	5 (4.2%)	9 (7.6%)	1.87 (0.61–5.74)
I sought support to process the EAS			
No	79 (65.8%)	69 (58.0%)	Reference
Yes	41 (34.2%)	50 (42.0%)	1.40 (0.83–2.36)

^a^ Missings varied between 5 and 12 (2.0–4.9%). Bold indicates statistical significance (*p* < 0.05)

## Discussion

This study showed that EAS requests have a large impact on physicians. Concerns about specific aspects of the EAS process, such as the emotional burden of preparing and performing EAS, were commonly reported by physicians. Amongst physicians who refused a request, a substantial number experienced pressure to grant the request. Especially general practitioners who were 55 years or older felt pressured by the patient to grant the request, more so if the patient was older than 80 years, had a life-expectancy of 6 months or more and did not have cancer. The large majority of physicians who performed EAS reported contradictory emotions afterwards. Older physicians who had little experience with euthanasia requests, who experienced pressure and who were concerned about administering lethal drugs were more likely to report burdensome feelings after performing EAS.

### Concerns about the EAS process

The results show that concerns about the euthanasia process are common amongst physicians who receive EAS requests. Concerns about the emotional burden of preparing EAS and performing EAS were reported by around 50% of both the physicians who reported on a refused request and the physicians who reported on a granted request. Physicians who refused a request were more likely to dread assessing the due care criteria and dealing with the relatives of the patient compared to physicians who granted a request. Previous research has shown that relatives may have difficulties with understanding and accepting the decision of the physician to refuse the request, which can be a reason for concern for physicians who refuse a request [15]. Although, only 0.2% of all EAS cases are judged to be not conform the due care criteria, a substantial number of physicians who granted a request dreaded the administrative burden, and found waiting for the judgment of the euthanasia review committee burdensome [9]. Concerns were common amongst those who reported on a granted request, but there was a relatively high frequency of concerns amongst those who reported on a refused request. This may be an indication that these requests were more complex [9, 16]. However, it is also possible that physicians who experienced more concerns, were more inclined to refuse the request.

### Pressure to grant the request

Literature from the past 5 years confirms our findings that physicians may experience pressure to perform EAS from the patient (29%) and the relatives (34%), and pressure to perform EAS as soon as possible (44%) [9]. Our study adds to this general observation by demonstrating that pressure is experienced more frequently when requests are refused than when they are granted. It is, however, unknown whether physicians refused the request because of the pressure by patients or relatives or whether physicians felt pressured because they refused the request.

The results from a recent interview study by Snijdewind et al. indicate that pressure experienced by physicians frequently stems from a difference in or unrealistic expectations: patients and relatives often do not understand why physicians are not (yet) willing to grant the request [15]. A lot of confusion seems to be caused by the written advance euthanasia directive. For example, the large majority of the general public and patients’ relatives is of the opinion that incompetent patients with advanced dementia should be able to receive EAS on the basis of a written advance euthanasia directive [17–19]. Few physicians, however, are willing to carry out EAS on the basis of such a directive [20]. Unmet expectations like these may lead to disappointed patients and this may contribute to the pressure physicians experience [21–23].

The association between the physician being older and experiencing pressure seems to contradict evidence that physicians who have more experience with providing EAS are better able to withstand pressure from patients [24]. There is, however, also evidence showing that performing EAS does not become easier for physicians who have more experience with it [25, 26]. It is possible that the increasing number of EAS requests from patients who do not suffer from life-limiting illnesses increases pressure [27, 28].

### Feelings after performing EAS

Seventy eight percent of the physicians who reported on a granted request reported uncomfortable feelings (the act felt burdensome, as a heavy responsibility and had emotional impact) after having performed EAS. Simultaneously, 67% reported comfortable feelings (satisfaction and relief). The simultaneous occurrence of these seemingly contradictory feelings shows the impact these requests have [24, 29]. The positive feelings physicians experience may be related to the fact that EAS is can be seen as an act of beneficence. The physician’s willingness to perform EAS can, therefore, be viewed as a supreme final act of care for the patient, that is associated with positive emotions such as satisfaction and relief. At the same time, the act can evoke negative emotions because of its extraordinary nature [24, 29, 30]. A significant association was found between burdensome feelings and age. Physicians aged between 40 and 54 were more likely to perceive performing EAS as burdensome compared to physicians aged 39 or younger. This may be explained by the fact that the younger physicians were trained in a context where euthanasia was legally regulated and, therefore, have a stronger tendency to regard it as ‘normal’. However, this does not explain yet why the oldest group of physicians (55 years or older) were equally likely to experience performing EAS as burdensome as the youngest group.

Over the past 20 years, ambiguity about performing EAS appears to have increased. The percentage of physicians reporting uncomfortable feelings after performing has remained largely stable: 72% in 1995 versus 80% in 2016, although the percentage of physicians reporting emotional feelings increased, from 30 to 44%. The percentage of physicians who reported comfortable feelings after having performed EAS increased from 54% in 1995 to 67% in 2016 [10]. This increase is mainly attributed to an increase in the percentage of physicians who reported feelings of satisfaction, which increased from 46 to 60%.

The percentage of physicians who reported uncomfortable feelings is higher for those who performed EAS compared to those who took other end-of-life decisions. Of physicians who administered opioids to alleviate pain or other symptoms in doses which the physician believed to be large enough to have a probable life-shortening effect, only 18% reported uncomfortable feelings including the act being burdensome (7%), emotional (11%) and a heavy responsibility (6%), whereas of physicians who performed EAS, 80% reported uncomfortable feelings including the act being burdensome (50%), a heavy responsibility (56%) and emotional (44%) [10]. This difference can be explained by the direct, causal relation between the act of EAS and the death of the patient which may be experienced as unnatural.

### Strengths & limitations

The most important strength of this study is the nationwide sample of physicians working in different specialties. In addition, stratification for outcome of the request had several advantages. First, it provides an unique insight into the characteristics of refused EAS requests, on which literature is scarce. Second, by stratifying physicians to either answer questions about a refused or a granted request, we ensured that there were a sufficient number of cases for both outcomes of the requests.

A possible limitation is selection bias. The response was relatively low, mainly because of a low response amongst medical specialists (37%). Non-responders might differ from responders in their views on the impact of EAS requests. Another possible limitation is recall bias. Physicians were asked about concerns, pressure and emotions experienced in the most recent case in which they refused or granted a request. People may remember events differently from what really happened and memories may change over time. Furthermore, by asking physicians to describe a request from a patient who did not have cancer, there might be an overestimation of experienced pressure. Lastly, a power problem in the group of physicians who reported on a granted case may be the reason for the absence of significant associations for perceived pressure. Despite these limitations, this study provides valuable insights in the impact of EAS requests on physicians about which literature is scare.

## Conclusion

EAS requests have a substantial impact on physicians, whether they are willing to grant the request or not. This is not necessarily problematic, but it is nonetheless important to be aware of this. There are indications of an overall tendency of EAS becoming increasingly ‘normal practice’ in the eyes of the general public [23]. This is expressed in patients sometimes claiming EAS, i.e. patients increasingly considering EAS a right. Indeed, a recent study showed that the percentage of the general public who are of the opinion that people should have the right to receive EAS increased, from 57% in 2010 to 67% in 2016 [9]. As a result, physicians may experience less room for a careful decision making process and they may even feel forced to cross their own moral boundaries or be tempted to perform euthanasia and assisted suicide in cases that may not meet the criteria. However, there is also anecdotal evidence that physicians become less willing to grant an EAS request as a result of pressure. It is important that society becomes aware of the impact EAS requests have on physicians. Moreover, there should be attention for the tension physicians may experience resulting from a discrepancy between society’s expectations and physician’s willingness to perform EAS.

## Data Availability

The datasets used and/or analyzed during the current study are available from the corresponding author on reasonable request.

## Abbreviations

CI: Confidence Intervals
EAS: Euthanasia and/or assisted suicide
OR: Odds ratios
